# Error in Results

**DOI:** 10.1001/jamanetworkopen.2022.35316

**Published:** 2022-09-15

In the Original Investigation titled “Efficacy of a Commercial Weight Management Program Compared With a Do-It-Yourself Approach: A Randomized Clinical Trial,”^1^ published August 16, 2022, there was an error in the second paragraph of the Secondary Outcomes subsection of the Results section. The name of the measure (ie, quality of life) was inadvertently deleted. It should read as follows: “At 3 months, participants in the commercial program group had significantly greater improvements than those in the DIY group in quality of life (total score, 2.49 [97.5% CI, 0.03-4.95]; *P* = .02; and physical function subscale, 4.10 [97.5% CI, 1.08-7.13]; *P* = .02).” This article has been corrected.^1^
